# Prolonged Postoperative Pyrexia and Transient Nonnephrogenic Vasopressin-Analogue-Resistant Polyuria following Endoscopic Transsphenoidal Resection of an Infundibular Epidermoid Cyst

**DOI:** 10.1155/2021/6690372

**Published:** 2021-04-13

**Authors:** Yuichiro Yoneoka, Yasuhiro Seki, Katsuhiko Akiyama, Yuki Sakurai, Nobumasa Ohara, Go Hasegawa

**Affiliations:** ^1^Department of Neurosurgery, Uonuma Kikan Hospital, Uonuma Institute of Community Medicine, Niigata University Medical and Dental Hospital, Niigata 949-7302, Japan; ^2^Department of Endocrine, Uonuma Kikan Hospital, Uonuma Institute of Community Medicine, Niigata University Medical and Dental Hospital, Niigata 949-7302, Japan; ^3^Department of Pathology, Uonuma Kikan Hospital, Uonuma Institute of Community Medicine, Niigata University Medical and Dental Hospital, Niigata 949-7302, Japan

## Abstract

Prolonged postoperative pyrexia (PPP) due to Mollaret's meningitis following endoscopic transsphenoidal surgery (eTSS) for an intracranial epidermoid cyst can be confused with postoperative meningeal infection after transsphenoidal resection, especially in the middle of the COVID-19 pandemic. Anosmia, as well as dysgeusia, cannot be evaluated in patients of eTSS for a while after surgery. We report a case of an infundibular epidermoid cyst with post-eTSS Mollaret's meningitis (MM). The post-eTSS MM caused vasopressin-analogue-resistant polyuria (VARP) in synchronization with PPP. A 59-year-old man experiencing recurrent headaches and irregular bitemporal hemianopsia over three months was diagnosed with a suprasellar tumor. The suprasellar tumor was an infundibular cyst from the infundibular recess to the posterior lobe of the pituitary, which was gross-totally resected including the neurohypophysis via an extended eTSS. Since awakening from general anesthesia after the gross total resection (GTR) of the tumor, the patient continuously had suffered from headache until the 13^th^ postoperative day (POD13). The patient took analgesics once a day before the surgery and three times a day after the surgery until POD11. Pyrexia (37.5–39.5 degree Celsius) in synchronization with nonnephrogenic VARP remitted on POD18. Intravenous antibiotics had little effect on changes of pyrexia. Serum procalcitonin values (reference range <0.5 ng/mL) are 0.07 ng/mL on POD12 and 0.06 ng/mL on POD18. His polyuria came to react with sublingual desmopressin after alleviation of pyrexia. He left the hospital under hormone replacement therapy without newly added neurological sequelae other than hypopituitarism. After GTR of an infundibular epidermoid cyst, based on values of serum procalcitonin, post-eTSS MM can be distinguished from infection and can be treated with symptomatic treatments. The postoperative transient nonnephrogenic VARP that differs from usual central diabetes insipidus can react with sublingual desmopressin after alleviation of PPP in the clinical course of post-eTSS MM. An infundibular epidermoid cyst should be sufficiently resected in one sitting to minimize comorbidities, its recurrence, or postoperative MM to the utmost.

## 1. Introduction

Mollaret's meningitis, a benign recurrent aseptic disease, is known to be associated with intracranial and intraspinal epidermoid cysts [1–7]. In practice, Mollaret's meningitis following resection of an intracranial epidermoid cyst can be confused with postoperative bacterial meningitis, especially after transsphenoidal surgery (TSS). We report a case of an infundibular epidermoid cyst with a hard-to-control polyuria in synchronization with prolonged postoperative pyrexia (PPP) during the postoperative course coincidentally in the middle of the coronavirus disease 2019 (COVID-19) pandemic. This report provides insight into the differential diagnosis of PPP from bacterial/viral meningitis and insight into coping to synchronized hard-to-control polyuria (vasopressin-analogue-resistant polyuria, which is described below) that differs from usual central diabetes insipidus (DI). For future reference, we describe these rare clinical manifestations in this report.

## 2. Case Report

**Figure 1:**
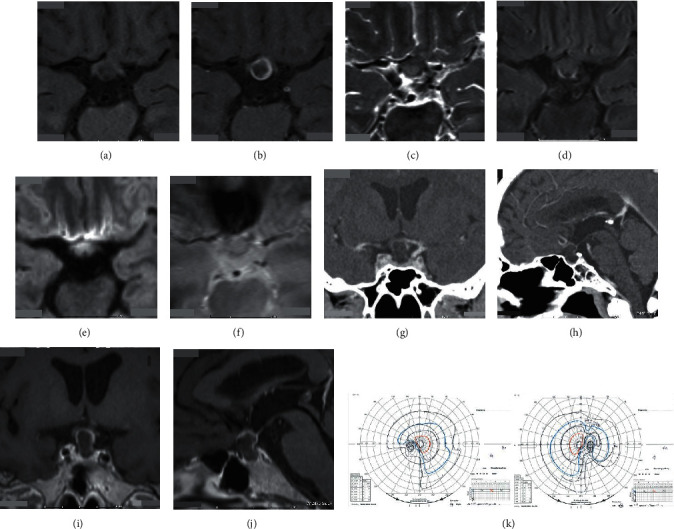
Precontrast T1-weighted axial image showing a suprasellar tumor compressing the optic pathway (a). Gadolinium-enhanced axial image showing an enhanced rim of the tumor (b). The contents of the tumor are of low signal intensity on the T1-weighted image (a) and Gadolinium-enhanced image (b), slightly high on T2-weighted image (c), iso on fluid-attenuated inversion recovery image (d), and relatively high on diffusion-weighted image (e) and T2∗-weighted image (f). Contrast-enhanced computed tomography showing a cystic lesion (g) from the sella turcia to the suprasellar space (h). Calcified components are not found in it. Postcontrast magnetic resonance imaging shows the enhanced cystic wall displacing the optic chiasm upward (i). This cystic lesion occupies the suprasellar region but not the third ventricle (h), (j). Goldmann perimeter reveals irregular bitemporal hemianopsia (k). Constriction of the visual field is more predominant in the right eye than in the left eye (k).

A 59-year-old man presented with a three-month history of gradually progressive blurred vision with headaches (mainly retrobulbar pain). The headaches were frequently associated with coughs or sneezes. Magnetic resonance (MR) imaging demonstrated a sellar-suprasellar tumor compressing the optic chiasm superiorly (Figures 1(a)–1(f)). Postcontrast computed tomography (CT) showed the enhanced rim of the sellar-suprasellar tumor without apparent calcification (Figures 1(g) and 1(h)). Postcontrast MR images demonstrated the enhanced rim of the tumor, indicating that the tumor was a cystic lesion with some contents (Figure 1(b)). Reconstructed CT and MR images revealed that the cyst corresponded to the pituitary stalk (Figures 1(g)–1(j)). Goldmann perimeter (GP) revealed irregular bitemporal hemianopsia predominantly in the left eye (Figure 1(k)). A closed interview detected the patient's recent polyuria and polydipsia. His headaches were associated with visual blurring. These symptoms got worse, and he decided to undergo an endoscopic transsphenoidal surgery (eTSS) for resection of the cyst. Preoperative diagnosis was craniopharyngioma, Rathke's cleft cyst, or sellar-suprasellar epidermoid cyst. On admission, the patient's body temperature was 36.4 degree Celsius (°C). A chest CT scan did not show pneumonia, pneumonitis, nor ground glass opacities, but clear lung fields. Complete blood count revealed neither lymphocytopenia nor thrombocytopenia. The patient had not experienced anosmia nor dysgeusia. He did not have any high-risk contact with COVID-19-infected persons.

**Figure 2:**
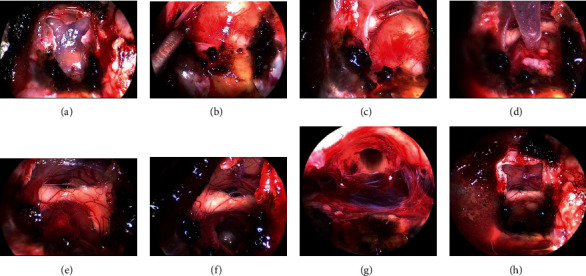
The intercavernous sinus has been cut after cauterization (a). A Y-shaped dural incision is made (a). The cyst wall is found above the pituitary gland (b). Vascular streaks are found on the cyst wall, indicating the pituitary stalk is tumorized (b). The cyst is displacing the optic chiasm (c). The cyst contents are aspirated via a long needle (d). After the aspiration (d), the cyst is shrunken so that the working space in this tumor resection is secured. An indentation is observed in the inferior surface of the decompressed optic chiasm (e). The solid cyst contents appeared as tissue debris, keratin, solid cholesterol, and their mixtures, which were meticulously removed (f). Since the cyst wall is the tumorized pituitary stalk, the cyst was gross-totally removed from the intrasellar part to the infundibular part (g). The opened skull base (h) is reconstructed with a fat-on-fascia graft plug [8].

**Figure 3:**
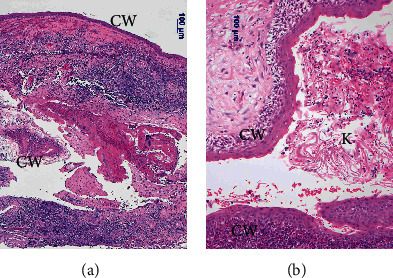
Histopathological examination shows features of the dermoid cyst. The lining is typically a squamous epithelium adjacent to glial tissues with inflammation (a). The cyst lumen contains keratin (b). CW: cyst wall and K: keratin.

**Figure 4:**
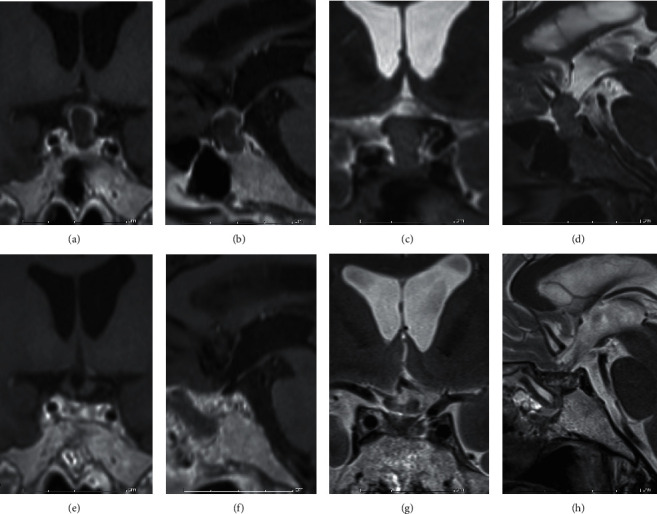
The displaced optic chiasm (a, b, c, and d) is successfully decompressed, and the cyst is totally resected (e, f, g, and h) on MR imaging at 4 months (19 weeks) weeks after surgery.

Under general anesthesia, an endoscopic transsphenoidal surgery was performed. The sellar floor and the adjacent tuberculum sellae were removed to expose the dura around the sella turcica. The dura was opened (Figure 2(a)) to expose the tumor and the pituitary gland (Figures 2(b) and 2(c)). Prior to the resection, the cyst was punctured with a long needle (Figure 2(d)). Some of the orange-xanthochromic mucus were aspirated. After the cyst aspiration (Figure 2(e)), not to disseminate the cyst contents for prevention of chemical meningitis, the cyst was opened and dissected (Figure 2(f)). The solid cyst contents appeared as tissue debris, keratin, and solid cholesterol, and their mixtures were meticulously removed. The cyst wall was carefully detached from the optic chiasm. Since the cyst wall was the tumorized pituitary stalk, the cyst was gross-totally removed from the intrasellar part to the infundibular part (Figure 2(g)). The opened skull base (Figure 2(h)) was reconstructed with a fat-on-fascia graft plug [8]. Corticosteroid (hydrocortisone) was administrated continuously because of total hypophysectomy after operation. Since awakening from general anesthesia after the gross total resection (GTR) of the tumor, the patient continuously suffered from headaches until the 13^th^ postoperative day (POD13). The patient took analgesics once a day before the surgery and three times a day after the surgery until POD11. The patient's diabetes insipidus (DI) became stronger after the eTSS. This DI was controlled with sublingual desmopressin tablets until POD11. Pyrexia did not emerge until the patient's peroral analgesic dose decreased on POD11. This pyrexia (37.5–39.5°C) continued until POD18. This prolonged postoperative pyrexia (PPP) could be controlled with peroral nonsteroidal antiinflammatory drugs (NSAIDs). In addition, hard-to-control polyuria (4730 ± 1361 ml, mean ± SD), in other words, vasopressin-analogue-resistant polyuria (VARP), emerged in synchronization with PPP and continued with some alterations until POD35. This PPP was unresponsive to intravenous pitressin or sublingual desmopressin tablets. The plasma natrium fluctuated from 123 mEq/L to 135 mEq/L, which was below the normal range (138 mEq/L–145 mEq/L), during the VARP. Water supply and sodium correction were performed on the basis of laboratory data. Although his nuchal stiffness was not observed, his neck was not supple retrospectively. Although anosmia and dysgeusia could not be estimated because of postoperative nasal packing, leukocytopenia was not found in the patient [9]. Intravenous antibiotics had little effect on changes of pyrexia. Serum procalcitonin values (reference range < 0.5 ng/mL) were 0.07 ng/mL on POD12 and 0.06 ng/mL on POD18. Based on values of serum procalcitonin, we provided him with symptomatic treatments in addition to prophylactic antibiotics: piperacillin and amikacin. His pyrexia remitted on POD18. After POD18, he did not suffer from headaches. His polyuria began to react with sublingual desmopressin after alleviation of pyrexia and turned into usual DI after 5 weeks of the eTSS. The plasma natrium was normalized. His corrected vision was 20/16 in both eyes despite his bitemporal hemianopsia not improving sufficiently. The patient left the hospital without new sequalae except for postoperative panhypopituitarism under hormone replacement therapy including peroral hydrocortisone, levothyroxine, and desmopressin. Histopathological analysis confirmed the cyst as an epidermoid cyst (Figure 3). In fact, we had encountered a dearth of coronavirus testing kits during his perioperative care in the middle of the COVID-19 pandemic. MR imaging at 4 months (19 weeks) after surgery showed that the displaced optic chiasm (Figures 4(a)–4(d)) had been successfully decompressed and the infundibular dermoid cyst was totally resected (Figures 4(e)–4(h)).

## 3. Discussion

Only six cases of infundibular epidermoid cysts were reported prior to this report (Table 1) [10–15]. In fact, infundibular epidermoid cysts are rare, and surgical experiences are valued.

From this case, we have found two things that the previous reports did not refer to [10–15]; the first is postoperative prolonged pyrexia (PPP), and the second is postoperative transient vasopressin-analogue-resistant polyuria (VARP).

### 3.1. Postoperative Prolonged Pyrexia (PPP)

The PPP should be distinguished from a symptom of postoperative bacterial meningitis. Because healthcare-associated meningitis or ventriculitis is a serious complication in different neurosurgical procedures and is associated with significant morbidity and mortality [16], in the middle of the COVID-19 pandemic, the PPP should also be distinguished from COVID-19 infection [17].

Fever is a relatively common occurrence among patients in the intensive care setting. Although the most obvious and concerning etiology is sepsis, drug reactions, venous thromboembolism, and postsurgical fevers are all on the differential diagnosis [18]. In the middle of the COVID-19 pandemic, not only sepsis but also COVID-19 infection is a nonnegligible differential diagnosis [19], as well as postoperative meningitis. There is abundant evidence that fever is detrimental in acute neurologic injury [18]. Raised temperature may be due to a regulated readjustment in the hypothalamic “set point” in response to inflammation and infection, or it may occur as a consequence of damage to the hypothalamus and/or its pathways [20]. This primarily occurs in SAH and TBI, with hypothalamic injury being the proposed mechanism. Paroxysmal sympathetic hyperactivity is another source of hyperthermia commonly seen in the population with traumatic brain injury [18]. In the brain, the preoptic area and the paraventricular hypothalamus are part of a neuronal network mediating sympathetic activation underlying fever [21]. Thus, it is no wonder that infundibular epidermoid cysts cause central hyperthermia following chemical meningitis that is known as Mollaret's meningitis [1–7]. Ganko et al. reported level II evidence that establishes the efficacy of prophylactic steroids utilized in patients undergoing surgery for epidermoid cysts to prevent postoperative chemical meningitis [22].

Serum procalcitonin levels seem to be the best marker in differentiating between bacterial and viral meningitis in adults [23]. In addition, serum procalcitonin might be able to discriminate between bacterial and chemical causes of meningitis in postmyelographic meningitis [24]. In our case, after GTR of the infundibular epidermoid cyst, the patient's pyrexia with headaches was diagnosed as postoperative Mollaret's meningitis following the infundibular epidermoid cyst that was treated with symptomatic treatments based on values of serum procalcitonin [24, 25]. The patient's serum procalcitonin values (reference range < 0.5 ng/mL) were 0.07 ng/mL on POD12 and 0.06 ng/mL on POD18. In addition, a nuchal rigidity (stiff neck) was unobserved as well as nausea in the patient, and his level of consciousness was 15 on the Glasgow Coma Scale even with a fever greater than 38°C [26–28]. Serum procalcitonin is a useful tool in the evaluation of patients with a known or suspected central nervous system infection [25].

### 3.2. Postoperative Hard-To-Control Polyuria: Vasopressin-Analogue-Resistant Polyuria

The postoperative hard-to-control polyuria: pitressin/desmopressin-resistant polyuria, which should instead be called vasopressin-analogue-resistant polyuria (VARP), differs from usual central diabetes insipidus (DI). VARP came to react with sublingual desmopressin after alleviation of pyrexia in the clinical course of post-eTSS Mollaret's meningitis in our case. The postoperative VARP is something different from usual DI: hypernatremia associating polyuria was not observed, indicating excessive excreted urinary natrium quantity during VARP. The VARP interacts with central hyperthermia (PPP), which suggests an involvement of the abovementioned hypothalamic dysfunction during the transient VARP. This VARP may belong to the category of cerebral salt wasting syndrome coexisting with DI [29], or is VARP a new clinical entity? Further studies are warranted to answer this question.

### 3.3. Transient Hypothalamic Dysfunction Is Inflammation-Induced or Trauma-Induced?

PPP in this case was linked with the patient intake of NSAIDs. The VARP settled as the PPP remitted. Based on these findings and the clinical course, transient hypothalamic dysfunction (HD) is probably inflammation-induced. If so, NSAIDs and corticosteroids might be effective for HD after resection of infundibular epidermoid cysts.

### 3.4. Resection of Infundibular Epidermoid, Gross Total, or Partial?

This case is the first report of transient nonnephrogenic VARP synchronizing with PPP during Mollaret's meningitis after resection of an infundibular epidermoid cyst. As a similar mechanism, Rathke's cleft cysts (RCCs) sometimes present with acute onset, and the presentation is consistent with the features of pituitary apoplexy caused by pituitary adenoma [30]. As well, RCCs may cause Mollaret's meningitis and pituitary failure [31]. Because the infundibular epidermoid is close to the pituitary gland and hypothalamus, the infundibular epidermoid cyst and its contents should be sufficiently resected in one sitting, as much as possible within safe limits. According to a meta-analysis by Shear et al. [32], subtotal resection is the primary risk factor for cyst recurrence, which can occur many years after the initial surgery. In the meta-analysis of observational studies in 691 patients, the pooled rates of recurrence after gross total and subtotal resection were 3 and 21%, respectively [32]. The average recurrence rate for studies with longer follow-up durations (≥4.4 years) (17.4%) was significantly higher than the average recurrence rate for studies with shorter follow-up durations (<4.4 years) (5.7%) [32]. A recurrence rate of 21% in subtotal resection of an epidermoid should not be ignored. Thus, gross total resection is the primary treatment goal of an intracranial epidermoid, if possible. Among the previous reports of infundibular epidermoid cysts [10–15], not all epidermoid cysts were resected gross-totally. Based on the recurrence rate of 21% in subtotal resection of epidermoid [32], careful and long-term follow-up is necessary even after subtotal resection.

In our case, even under GTR of an infundibular epidermoid cyst, a post-eTSS Mollaret's meningitis continued until POD18 and had settled after POD19. If the eTSS resulted in insufficient resection, the Mollaret's meningitis would not have settled and been long lasting. Thus, surgical gross total resection remains the mainstay for treatment of intracranial epidermoid cysts [29, 33]. Here, again, an infundibular epidermoid cyst should be sufficiently resected in one sitting, as much as possible within safe limits.

In the middle of the COVID-19 pandemic, prolonged pyrexia should be appropriately diagnosed and distinguished from coronavirus infection or other diseases. Physicians need to correctly approach this new disease in daily clinical practice, often representing a challenge in terms of differential diagnosis [34].

## 4. Conclusions

After GTR of the infundibular epidermoid cyst, based on values of serum procalcitonin, post-eTSS Mollaret's meningitis can be distinguished from infection and can be treated with symptomatic treatments despite PPP. The postoperative VARP differs from usual central DI and can react with sublingual desmopressin after alleviation of PPP in the clinical course of post-eTSS Mollaret's meningitis. An infundibular epidermoid cyst should be sufficiently resected in one sitting on the basis of its recurrence rate.

## Figures and Tables

**Table 1:** Characteristics of previous and current case reports of pituitary stalk epidermoid cysts.

Paper	Age/sex	Presentation	Imaging characteristics	Operative approach	Postoperative status
Costa et al., 2013 [10]	27-year-old female	Amenorrhea, galactorrhea, polyuria, and polydipsia	Mixed signal, bilobed rim and enhancing cystic lesion	Endoscopic endonasal extended transsphenoidal	Not reported

Nakassa et al., 2017 [11]	54-year-old female	Headache, visual disturbance, polyuria, and polydipsia	Mixed signal and nonenhancing cystic lesion	Endoscopic endonasal	Persistent DI and subjective visual fields improvement

McCormack et al., 2018 [12]	36-year-old female	Headache and visual disturbance	Multilocular T1 hypointense, T2 hyperintense, and rim-enhancing lesion	Endoscopic endonasal extended transsphenoidal	Transient DI

Montaser et al., 2018 [13]	49-year-old female	Headache	Mixed signal and nonenhancing sellar/suprasellar cyst extending into the third ventricle	Endoscopic endonasal extended transsphenoidal	Persistent DI

Khan et al., 2019 [14]	55-year-old male	Decreased visual acuity	Mixed signal and rim-enhancing cystic lesion	Endoscopic endonasal extended transsphenoidal	Panhypopituitarism and subjective visual fields improvement

Lee et al., 2020 [15]	63-year-old male	Polydipsia and polyuria	T2 hyperintense and rim-enhancing cystic lesion	Pretemporal craniotomy	Persistent DI

Current case	59-year-old male	Headache and visual disturbance	Rim-enhancing cystic lesion	Endoscopic endonasal extended transsphenoidal	Panhypopituitarism, persistent DI, and visual fields improvement

## Data Availability

No data were used to support this study.

## References

[1] Chadarévian J.-P. D., Becker W. J. (1980). Mollaretʼs recurrent aseptic meningitis. *Journal of Neuropathology and Experimental Neurology*.

[2] Szabó M., Majtényi C., Guseo A. (1983). Contribution to the background of Mollaret’s meningitis. *Acta Neuropathologica*.

[3] Becker W. J., Watters G. V., Chadarevian J.-P. d., Vanasse M. (1984). Recurrent aseptic meningitis secondary to intracranial epidermoids. *Canadian Journal of Neurological Sciences/Journal Canadien des Sciences Neurologiques*.

[4] Crossley G. H., Dismukes W. E. (1990). Central nervous system epidermoid cyst: a probable etiology of Mollaret’s meningitis. *The American Journal of Medicine*.

[5] Achard J.-M., Lallement P.-Y., Veyssier P. (1990). Recurrent aseptic meningitis secondary to intracranial epidermoid cyst and Mollaret’s meningitis: two distinct entities or a single disease? a case report and a nosologic discussion. *The American Journal of Medicine*.

[6] Aristegui F. J., Delgado R. A., Oleaga Z. L., Hermosa C. C. (1998). Mollaret’s recurrent aseptic meningitis and cerebral epidermoid cyst. *Pediatric Neurology*.

[7] Gao B., Yang J., Zhuang S. (2007). Mollaret meningitis associated with an intraspinal epidermoid cyst. *Pediatrics*.

[8] Yoneoka Y., Aizawa N., Nonomura Y., Ogi M., Seki Y., Akiyama K. (2020). Traumatic nonmissile penetrating transnasal anterior skull base fracture and brain injury with cerebrospinal fluid leak: intraoperative leak detection and an effective reconstruction procedure for a localized skull base defect especially after coronavirus disease 2019 outbreak. *World Neurosurgery*.

[9] Meng X., Deng Y., Dai Z., Meng Z. (2020). COVID-19 and anosmia: a review based on up-to-date knowledge. *American Journal of Otolaryngology*.

[10] Costa F., Fornari M., Felisati G., Maccari A., Bauer D., Lasio G. (2013). Epidermoid cyst of the pituitary stalk. *Neurosurgery Quarterly*.

[11] Nakassa A. C. I., Chabot J. D., Snyderman C. H., Wang E. W., Gardner P. A., Fernandez-Miranda J. C. (2017). Complete endoscopic resection of a pituitary stalk epidermoid cyst using a combined infrasellar interpituitary and suprasellar endonasal approach: case report. *Journal of Neurosurgery*.

[12] McCormack E. P., Cappuzzo J. M., Litvack Z., Almira-Suarez M. I., Sherman J. S. (2018). Suprasellar epidermoid cyst originating from the infundibulum: case report and literature review. *Cureus*.

[13] Montaser A., Revuelta Barbero J., Shahein M. (2018). Endoscopic endonasal transtuberculum sellae approach for the resection of suprasellar intrainfundibular epidermoid cyst. *Journal of Neurological Surgery Part B: Skull Base*.

[14] Khan A. B., Goethe E. A., Hadley C. C. (2019). Infundibular epidermoid cyst: case report and systematic review. *World Neurosurgery*.

[15] Lee P., Krisht K. M., Mukunyadzi P., Krisht A. F. (2021). Resection of an isolated pituitary stalk epidermoid cyst through a pretemporal approach: case report and review of the literature. *World Neurosurgery*.

[16] Li Y., Wang R., Song P.-X. (2020). Impact of an educational program on reducing health care-associated meningitis or ventriculitis in the neurosurgical intensive care unit. *American Journal of Infection Control*.

[17] Fleseriu M., Buchfelder M., Cetas J. S. (2020). Pituitary society guidance: pituitary disease management and patient care recommendations during the COVID-19 pandemic-an international perspective. *Pituitary*.

[18] Meier K., Lee K. (2017). Neurogenic fever. *Journal of Intensive Care Medicine*.

[19] Hasani H., Mardi S., Shakerian S., Taherzadeh-Ghahfarokhi N., Mardi P. (2020). The novel coronavirus disease (COVID-19): a PRISMA systematic review and meta-analysis of clinical and paraclinical characteristics. *BioMed Research International*.

[20] Childs C. (2008). Human brain temperature: regulation, measurement and relationship with cerebral trauma: part 1. *British Journal of Neurosurgery*.

[21] Griton M., Konsman J. P. (2018). Neural pathways involved in infection-induced inflammation: recent insights and clinical implications. *Clinical Autonomic Research*.

[22] Ganko R., Rodriguez M., Magnussen J., Simons M., Myint E., Assaad N. (2020). Do prophylactic steroids prevent chemical meningitis in surgery for epidermoid cysts? case report and literature review. *Surgical Neurology International*.

[23] Viallon A., Zeni F., Lambert C. (1999). High sensitivity and specificity of serum procalcitonin levels in adults with bacterial meningitis. *Clinical Infectious Diseases*.

[24] Bender A., Elstner M., Paul R., Straube A. (2004). Severe symptomatic aseptic chemical meningitis following myelography: the role of procalcitonin. *Neurology*.

[25] Velissaris D., Pintea M., Pantzaris N. (2018). The role of procalcitonin in the diagnosis of meningitis: a literature review. *Journal of Clinical Medicine*.

[26] Bijlsma M. W., Brouwer M. C., Kasanmoentalib E. S. (2016). Community-acquired bacterial meningitis in adults in The Netherlands, 2006-14: a prospective cohort study. *The Lancet Infectious Diseases*.

[27] van de Beek D., de Gans J., Spanjaard L., Weisfelt M., Reitsma J. B., Vermeulen M. (2004). Clinical features and prognostic factors in adults with bacterial meningitis. *New England Journal of Medicine*.

[28] Aronin S. I., Peduzzi P., Quagliarello V. J. (1998). Community-acquired bacterial meningitis: risk stratification for adverse clinical outcome and effect of antibiotic timing. *Annals of Internal Medicine*.

[29] Lee J. S., Baek H. J., Kim C. J., Yang E. M. (2020). Coexistence of central diabetes insipidus and prolonged cerebral salt wasting syndrome after brain tumor surgery: a case report. *Childhood Kidney Diseases*.

[30] Komatsu F., Tsugu H., Komatsu M. (2010). Clinicopathological characteristics in patients presenting with acute onset of symptoms caused by Rathke’s cleft cysts. *Acta Neurochirurgica*.

[31] Dancer C. M., Woods M. L., Henderson R. D., Robertson T., Mungomery M., Allworth A. (2008). Mollaret’s meningitis and pituitary failure associated with a Rathke’s cleft cyst. *Internal Medicine Journal*.

[32] Shear B. M., Jin L., Zhang Y. (2020). Extent of resection of epidermoid tumors and risk of recurrence: case report and meta-analysis. *Journal of Neurosurgery*.

[33] Rehman L., Bokhari I., Siddiqi S. U., Bagga V., Hussain M. M. (2018). Intracranial epidermoid lesions: our experience of 38 cases. *Turkish Neurosurgery*.

[34] Bertolino L., Vitrone M., Durante-Mangoni E. (2020). Does this patient have COVID-19? a practical guide for the internist. *Internal and Emergency Medicine*.

